# Preachers Who Sign Testimonials of Patent Nostrums and Secret Methods to Catch the Credulous

**Published:** 1890-07

**Authors:** 


PREACHERS WHO SIGN TESTIMONIALS OF PATENT NOSTRUMS AND SECRET METHODS TO CATCH THE CREDULOUS.—The alleged minister of the gospel of our Lord and Savior Jesus Christ who signs testimonials boosting up secret methods and patent humbug remedies, signs away all his rights to the claim of virtue, honesty and common decency; and procures instead thereof, a a bill of lading for a certain passage to the bottomless pit of everlasting hell fire and eternal damnation—world without end. Amen.

Preachers who thus stoop to this nefarious and groveling plane of humbuggery would be found, upon analysis, to be too infernally worthless to be classed fourth-rate soap grease; or, if pulverized with a buzz-saw, would not make as good manure as attenuated moonshine. But our vocabulary fails us in expressing their heinous crime of robbing widows and orphans; to say nothing of killing them outright by the various danger-stuffs they recommend; For the sake of gentility, morality, common decency, the church of Christ and His examples of charity while on earth—His suffering, death, burial, resurrection and ascension, we beg—earnestly beg—all ministers, deacons and church members, to cease bolstering up quack remedies by which the poor, ignorant and afflicted may be robbed daily and finally die from such stuff and be buried in a pauper's graveyard. For humanity's sake stop this. Be ashamed of yourselves!

To editors of religious newspapers we must say: You in advertising such stuff, become a party to this infamous crime; and unless you quit it the medical profession, scientific men all decent citizens will loose all confidence in, and respect for you—in other words you will make down right infidels of those you seek—or profess to seek—to save. We have you spotted and unless you cease this disgraceful conduct we will hold you before the world and shake you over the pit of Hell as you do sinners who are not half as vile and cussed mean as you are. Now, don't go round and lie about what we have said, like Wilford Hall, of New York, did, but tell the truth—this is addressed *alone* to ministers who sign testimonials of secrect medicines and advertise the same in the religious press. Statistics show that over 100,000 innocent babes are killed annually by these vile poisons. We have tried persuasion many times to stop ministers from such conduct, but all to no effect. Language is too weak to express the heinous crime of entering into a conspiracy to kill innocent babes at their mothers' breasts by all kind of poisonous soothing syrups; all of which contain opium, a very small fraction of a grain of which will kill an infant. Stop! We beg you to stop! For humanity's sake, for the sake of helpless infants, do not put your signature to a paper recommending a stuff of which you are ignorant of its composition.—*Texas Health Journal.*

